# Value of monitoring urine ammonia at time of biopsy in patients with lupus nephritis

**DOI:** 10.1186/s12882-020-02106-y

**Published:** 2020-10-20

**Authors:** Huanhuan Zhu, Huiting Wan, Suyan Duan, Chengning Zhang, Qing Li, Simeng Liu, Lin Wu, Bo Zhang, Changying Xing, Yanggang Yuan

**Affiliations:** Department of Nephrology, The First Affiliated Hospital of Nanjing Medical University, Nanjing Medical University, 300 Guangzhou Road, Nanjing, Jiangsu Province 210029 P.R. China

**Keywords:** Lupus nephritis, Urinary acidification function, Urine ammonia, Tubulointerstitial lesions

## Abstract

**Objective:**

Although lupus nephritis (LN) is mostly characterized by glomerular involvement, tubular injury is indispensable in its pathogenesis and progression. The purpose of this study is to examine associations between urinary acidification function and clinical and pathological features in LN.

**Methods:**

A total of 103 patients with renal biopsy-proven LN were included, and clinical parameters and laboratory data were obtained from the medical records. Plasma samples, 24-h urine samples and the urinary acidification function, including urine pH, titratable acid, and ammonia, were collected within 3 days before the day of renal biopsy. The correlations between defects of acid excretion and clinical and pathological features were then assessed. Logistic regression analysis was used to assess factors associated with the presence of nephrotic range proteinuria.

**Results:**

The urine ammonia level was inversely correlated with SLEDAI-2 K scores, rSLEDAI scores, serum creatinine levels and proteinuria, while it was positively correlated with eGFR. And urine titratable acid was only inversely correlated with rSLEDAI scores and proteinuria. Moreover, urine ammonia had significant negative correlations with AI scores, interstitial inflammatory cell infiltration, CI scores, glomerular sclerosis, fibrous crescents, tubular atrophy and interstitial fibrosis. And urine titratable acid was mainly inversely correlated with CI scores. Furthermore, univariate logistic analyses identified that both urine titratable acid and ammonia were correlated with the presence of nephrotic range proteinuria. After the adjustment for chronicity index and eGFR in a multivariate logistic analysis, only urine titratable acid was still identified as an independent risk factor for the occurrence of nephrotic range proteinuria.

**Conclusions:**

Urine ammonia was associated with clinical and pathological features of chronicity and tubulointerstitial disease activity among patients with lupus nephritis. Furthermore, the strong association between urinary protein and titratable acid excretion at the time of kidney biopsy is significant even after adjusting for the chronicity index and eGFR at biopsy.

## Background

Lupus nephritis (LN) carries high morbidity and mortality in patients with systemic lupus erythematosus (SLE), and 40% of patients with SLE will develop renal impairment for different degrees [1, 2]. The risk of end-stage kidney disease (ESRD) is high in lupus nephritis (up to 10%) [3]. Thus, accurate indicators are urgently needed to reflect the renal activity of LN and identify patients at high risks of progression to ESRD.

Unfortunately, conventional biomarkers such as serum creatinine levels, anti-dsDNA antibody titers, and complement levels perform poorly in predicting lupus renal flares and prognostic stratification [4]. Renal biopsy, the gold standard for diagnosis, represents the nature and severity of renal involvement to evaluate the risk of renal failure [5]. As an invasive diagnostic test, a repeat renal biopsy is unrealistic for longitudinally monitoring the response to therapy or the activity of lupus nephritis. Urinary biomarkers offer a window for assessing the response to treatment and the impending renal flare, which are excreted directly from the kidney and able to longitudinal monitoring. Proteinuria as the major candidate of urine presents a weak connection with the historical activity in LN and adverse renal outcomes [6]. There are several novel biomarkers including urinary angiostatin, vascular cell adhesion molecule 1, immunoglobulin binding protein 1, and the TNF-like weak inducer of apoptosis, which have been examined to reflect histological features of lupus nephritis and identify higher risk for renal outcome [7–10]. However, these biomarkers are still in its infancy and have not yet been widely used in clinical settings.

Renal tubular acidosis (RTA) is a defect of tubular function, which is characterized by the renal inability to reabsorb enough bicarbonate (HCO_3_^−^) to a proximal tubule or to secrete enough hydrogen (H^+^) through a distal tubule [11, 12]. The hydrogen is secreted into the final urine by the excretion of titratable acid and ammonia. There is an intimate connection between RTA and kidney disease such as diabetic nephropathy, tubulointerstitial nephropathies, and autoimmune disorder [13]. It is of note that renal acidification defects are associated with the risk of chronic kidney disease (CKD) progression. M Vallet et al. in the NephroTest cohort with CKD found urinary ammonia excretion decreased with the elevated glomerular filtration rate (GFR), which was associated with a significantly higher risk of kidney disease progression [14]. In diabetic nephropathy, patients exhibited a lower risk of progression toward ESRD in higher net acid excretion [15]. This is consistent with our previous study, which showed that defects of titratable acid and ammonia secretion were associated with GFR and the dysfunction of titratable acid secretion was an independent predictor for diabetic nephropathy progression [16]. It has been known that tubular lesions were common in lupus nephritis patients and correlated closely with renal outcomes [3, 17, 18]. In addition, RTA is a complication of lupus nephritis with a variety of tubular dysfunction and is intimately associated with disease activities [19].

Previous work has focused primarily on types of RTA and laboratory indices in LN. The role of urinary acidification function rather than overt metabolic acidosis in LN remains unknown. Therefore, the purpose of this study was to examine the association between defects of acid excretion and clinical and pathological features in LN.

## Methods

### Patients

A total of 103 patients with renal biopsy-proven lupus nephritis were recruited by the First Affiliated Hospital of Nanjing Medical University from January 2003 to October 2018 in this study. The inclusion criteria were as follows: (1) patients ≥18 years of age; (2) these fulfilled the 2012 SLE international collaborating clinics classification criteria [20]; (3) these with biopsy-proven lupus nephritis according to the International Society of Nephrology/Renal Pathology Society (ISN/RPS) in 2003 classification system [21]. And the exclusion criteria were as follows: (1) patients with other kidney diseases (such as minimal change disease and thrombotic microangiopathy) or diabetes; (2) these who took some sodium bicarbonate tablets or diuretics within 30 days. The ethical committee of the First Affiliated Hospital of Nanjing Medical University approved this study based on the Declaration of Helsinki. All participants signed written informed consent.

### Measurements

Clinical parameters and laboratory data were obtained from the medical records, including gender, age, SLE duration, blood pressure, serum creatinine, blood urea nitrogen (BUN), cystatin C, albumin, complement 3 (C3), antinuclear antibodies (ANA), anti-double-stranded DNA (anti-dsDNA), 24-h urine protein and so on. Plasma samples and 24-h urine samples were obtained from patients within 3 days before renal biopsy and before any immunosuppressive treatment. GFR was estimated using the chronic kidney disease epidemiology collaboration (CKD-EPI) equation [22]. The SLE disease activity index 2000 (SLEDAI-2 K) and renal SLE disease activity index (rSLEDAI) were used to assess SLE activity and kidney disease activity, respectively [7, 23]. The rSLEDAI consists of four parameters: hematuria, proteinuria, pyuria and urinary casts, each accounting for four scores. The evaluation of SLEDAI-2 K scores, rSLEDAI scores and the diagnosis of nephrotic syndrome were obtained 1 day before renal biopsy.

After 3 days of vegetarian diets, the urinary acidification function was detected from a fasting morning urine sample within 3 days before the day of renal biopsy, which was measured using a ZDJ-4B automatic potentiometric titrator (Shanghai INESA Scientific Instrument Co., Shanghai, China), as described in our previous study [16]. Normal values of the urinary acidification function are as follows: urine pH (5.0--8.0), titratable acid ≥10 mmol/L and ammonia ≥20 mmol/L.

### Renal histology

Renal biopsy specimens were examined by two experienced pathologists following the 2003 ISN/RPS classification system [21]. The activity index (AI) and chronicity index (CI) of lupus nephritis were assessed using the semi-quantitative scoring of the National Institutes of Health [24, 25]. Activity indices consisted of endocapillary proliferation, cellular crescents, fibrinoid necrosis, subendothelial hyaline deposits, interstitial inflammatory cell infiltration, and glomerular leukocyte infiltration. Chronicity indices consisted of glomerular sclerosis, fibrous crescents, tubular atrophy, and interstitial fibrosis. The segmental changes of indices were scored as follows: 0, normal; 1, < 25% of the acreage; 2, 25–50% of the acreage; and 3, > 50% of the acreage in each specimen. And glomerular sclerosis, cellular crescents, and fibrous crescents were calculated as percentages of the total number of glomeruli. The fibrinoid necrosis and cellular crescents were weighted by a factor of 2.

### Statistical analysis

All statistical analyses were evaluated using SPSS version 22 (SPSS, Chicago, IL, USA). Variables were presented as mean ± standard deviation, median (interquartile range), median with range (minimum, maximum) or percentage. Student’s t-test and one-way analysis of variance were used for data with a normal distribution. Mann-Whitney U test and Kruskal-Wallis test were used for data with a non-normal distribution. And the Chi-square test was used for qualitative variables. Spearman’s rank correlation was performed for correlation analysis of various lesions. Logistic analysis was performed to evaluate the association of urinary acidification function with nephrotic range proteinuria (24 h urinary protein > 3.5 g). The variables that showed a statistically significant association in univariate analysis were adjusted in a multivariate model. Results were expressed as odds ratios (OR) with 95% confidence intervals (95% CI). Statistical significance was considered as *p* < 0.05.

## Results

### Baseline characteristics of lupus nephritis

A total of 103 patients with lupus nephritis were included in this study. The clinical characteristics, laboratory parameters and pathological features of patients were shown in Table 1. The majority (83.5%) of the patients were female. The mean age was 39.75 ± 14.39 years, and the mean duration of SLE was 4 (1,24) months. The mean levels of eGFR, proteinuria, serum creatinine and C3 were 86.76 (52.11,117.36) ml/min/1.73 m^2^, 2.94 (1.29,5.51) g/24 h, 76.30 (53.70,116.20) umol/L and 0.49 (0.37,0.82) g/L, respectively. According to the ISN/RPS 2003 classification system, patient distribution by the pathological stages was as follows: class III, 14 (13.59%), class IV, 60 (58.25%) and class V, 29 (28.16%). The median levels of the activity indices and the chronicity indices were 4 (1,10) and 2 (0,11), respectively.

**Table 1:** Baseline characteristics in patients with lupus nephritis (*n* = 103)

Clinical evaluation		Laboratory assessment		Renal histopathology indices (median, range)	
Female,n(%)	86 (83.50)	WBC(10^9/L)	4.73 (3.50,7.06)	AI scores	4 (1,10)
Age (years)	39.75 ± 14.39	Hb(g/L)	100 (90,116)	Endocapillary hypercellularity	0 (0,1)
SLE duration (months)	4 (1,24)	PLT(10^9/L)	167.50 ± 67.34	Cellular crescents	0 (0,6)
Hypertension,n (%)	35 (33.98)	eGFR (ml/min/1.73 m^2^)	86.76 (52.11,117.36)	Karyorrhexis/fibrinoid necrosis	0 (0,2)
Hematuria,n(%)	54 (52.43)	Urine protein(g/24 h)	2.94 (1.29,5.51)	Subendothelial hyaline deposits	1 (0,2)
Nephrotic syndrome,n(%)	42 (40.78)	Cystatin C (mg/L)	1.57 (1.09,2.35)	Interstitial inflammatory cell infiltration	2 (1,3)
SLEDAI-2 K	11 (9,24)	SCr (umol/L)	76.30 (53.70,116.20)	Glomerular leukocyte infiltration	0 (0,1)
rSLEDAI	4 (4,8)	BUN (mmol/L)	6.93 (4.67,11.10)	CI scores	2 (0,11)
SBP (mmHg)	130 (122,143)	Uric acid (umol/L)	407.14 ± 125.09	Glomerular sclerosis	1 (0,3)
DBP (mmHg)	85 (78,91)	Albumin(g/L)	25.72 ± 7.39	Fibrous crescents	0 (0,3)
		Anti-ANA antibodies (+), n (%)	96 (93.20)	Tubular atrophy	1 (0,3)
		Anti-Sm antibodies(+), n (%)	54 (52.43)	Interstitial fibrosis	0 (0,3)
		Anti-dsDNA antibodies(+), n (%)	63 (61.17)		
		IgG(g/L)	12.75 (7.56,16.15)		
		C3(g/L)	0.49 (0.37,0.82)		
		C4(g/L)	0.10 (0.07,0.17)		

Values for categorical data were given as a number (percent); values for continuous variables were expressed as mean ± standard deviation (normally distributed data) or median (interquartile range) (non-normally distributed data); values for renal histopathology indices were expressed as median (minimum, maximum)

### Associations between urinary acidification function and clinical characteristics of lupus nephritis

Patients of lupus nephritis with nephrotic syndrome or nephrotic range proteinuria (> 3.5 g/24 h) had higher urine pH levels and lower urine titratable acid excretion. The level of urine ammonia was lower in patients with nephrotic range proteinuria. Urine ammonia excretion was less in males, whereas urine pH and titratable acid excretion did not differ between the sexes in lupus nephritis. There were no statistically significant differences in groups concerning age, hypertension, hematuria, and hypokalemia (Table 2).

**Table 2:** Associations of urinary acidification function with clinical and renal pathological features of lupus nephritis (*n* = 103)

	pH	Titratable acid (mmol/L)	Ammonia (mmol/L)
**Clinical data**			
Gender			
Male	6.15 ± 0.71	13 (8,16.75)	17 (11,26.25)
Female	6.11 ± 0.59	14 (8.5,18)	27 (16.5,40)
*p*	0.838	0.485	**0.015**
Age (years)			
< 30	6.01 ± 0.61	16 (9,23)	27 (18,38)
30–60	6.19 ± 0.61	13 (8.5,15.5)	26 (16,39)
> 60	6.10 ± 0.61	8 (5.5,15.5)	14 (10,28)
*p*	0.385	0.050	0.086
Hypertension			
Yes	6.00 ± 0.60	12.5 (9,15.25)	19.5 (12.5,31.75)
No	6.18 ± 0.61	14 (8,19)	32 (17,39)
*p*	0.156	0.433	0.051
Nephrotic syndrome			
Yes	6.30 ± 0.67	12.5 (7,16)	21.5 (13.25,35.75)
No	6.00 ± 0.54	14 (10,20)	27 (15.5,41)
*p*	**0.013**	**0.048**	0.168
SLEDAI-2 K			
r	0.077	−0.114	− 0.310
*p*	0.438	0.255	**0.002**
rSLEDAI			
r	0.216	− 0.374	− 0.445
*p*	**0.029**	**< 0.001**	**< 0.001**
**Laboratory data**			
Hematuria			
Yes	6.14 ± 0.58	14 (8,17)	24 (14.5,36.5)
No	6.09 ± 0.65	13.5 (8.75,21.5)	25.5 (15.75,41)
*p*	0.694	0.584	0.461
Hypokalemia			
Yes	6.21 ± 0.58	12.5 (7.5,15)	27 (14.25,36)
No	6.10 ± 0.62	14 (8,19)	24 (15.5,38.5)
*p*	0.513	0.420	0.802
SCr (umol/L)			
r	−0.256	−0.106	−0.500
*p*	**0.009**	0.293	**< 0.001**
eGFR (ml/min/1.73 m^2^)			
r	0.240	0.107	0.438
*p*	**0.015**	0.288	**< 0.001**
Urine protein (g/24 h)			
r	0.226	−0.301	−0.230
*p*	**0.022**	**0.002**	**0.021**
C3(g/L)			
r	0.020	−0.112	−0.125
*p*	0.848	0.276	0.224
Anti-dsDNA antibody drops			
r	−0.146	0.142	0.068
*p*	0.369	0.381	0.675
Nephrotic range proteinuria (> 3.5 g/24 h)			
Yes	6.28 ± 0.66	12 (7,16)	21.5 (13.25,35.75)
No	5.99 ± 0.54	15 (11,21.5)	27 (15.5,41.5)
*p*	**0.014**	**0.022**	**0.029**
**Pathological features**			
AI scores			
r	−0.191	−0.005	−0.212
*p*	0.054	0.961	**0.033**
Endocapillary hypercellularity			
r	−0.170	0.146	−0.027
*p*	0.087	0.146	0.790
Cellular crescents			
r	−0.133	0.058	−0.039
*p*	0.182	0.563	0.698
Karyorrhexis/fibrinoid necrosis			
r	0.028	−0.136	−0.083
*p*	0.778	0.176	0.411
Subendothelial hyaline deposits			
r	−0.075	−0.001	−0.090
*p*	0.449	0.995	0.372
Interstitial inflammatory cell infiltration			
r	−0.106	−0.181	− 0.377
*p*	0.288	0.070	**< 0.001**
Glomerular leukocyte infiltration			
r	−0.172	0.223	−0.008
*p*	0.082	**0.025**	0.940
CI scores			
r	0.167	−0.418	−0.474
*p*	0.091	**< 0.001**	**< 0.001**
Glomerular sclerosis			
r	0.061	−0.236	−0.345
*p*	0.543	**0.017**	**< 0.001**
Fibrous crescents			
r	−0.107	− 0.072	− 0.222
*p*	0.280	0.476	**0.025**
Tubular atrophy			
r	0.258	−0.447	− 0.457
*p*	**0.009**	**< 0.001**	**< 0.001**
Interstitial fibrosis			
r	0.166	−0.421	−0.419
*p*	0.095	**< 0.001**	**< 0.001**

Urine pH was positively correlated with rSLEDAI scores, eGFR, and proteinuria, whereas it was inversely correlated with serum creatinine levels. There were significantly negative correlations between urine titratable acid and rSLEDAI scores and proteinuria. Urine ammonia was inversely correlated with SLEDAI-2 K scores, rSLEDAI scores, serum creatinine levels, and proteinuria, while it was positively correlated with eGFR. There were no other differences between urinary acidification function and other clinical characteristics.

**Fig. 1:**
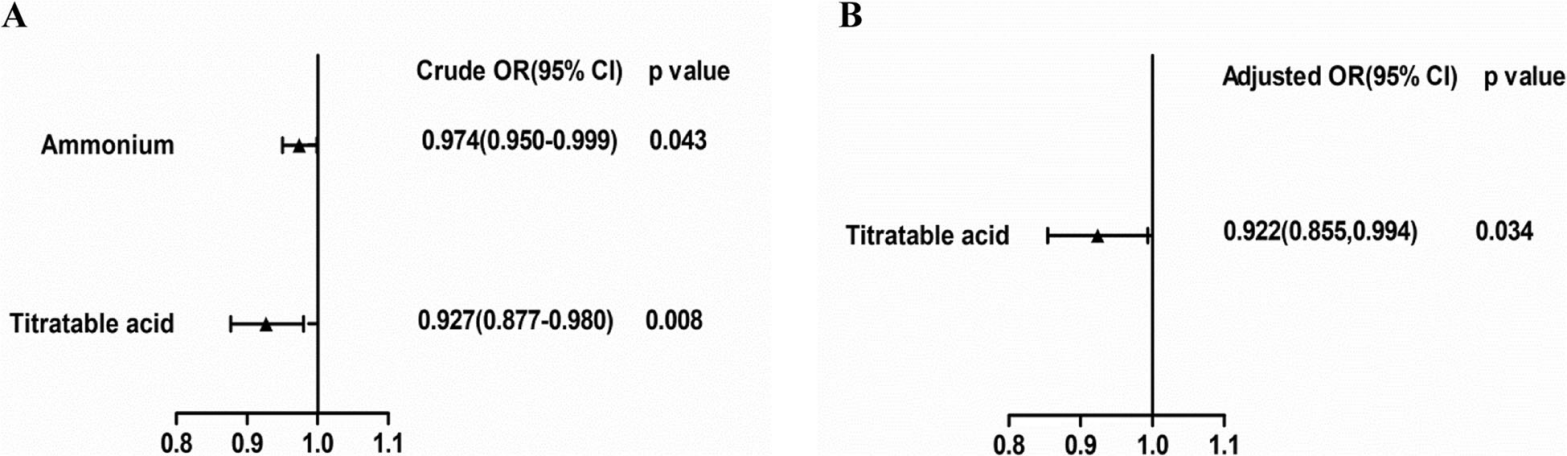
Logistic regression analysis of factors for distinguishing nephrotic range proteinuria. **a**. Titratable acid and ammonium were performed in a univariate logistic regression analysis. **b**. After the adjustment for chronicity index and eGFR, titratable acid was performed in a multivariate logistic regression analysis. Confidence intervals that do not cross the line of identity (1.0) are considered statistically significant. CI confidence interval; OR odds ratio

Moreover, univariate logistic regression analyses identified that both urine titratable acid and ammonia were correlated with the presence of nephrotic range proteinuria. After the adjustment for chronicity index and eGFR in a multivariate logistic regression analysis, urine titratable acid was still identified as an independent risk factor for the occurrence of nephrotic range proteinuria in patients with lupus nephritis. However, urine ammonia showed no association after the multivariable adjustment (Fig. 1).

### Associations between urinary acidification function and renal pathological features of lupus nephritis

As shown in Table 2, urinary acidification function did not associate with endocapillary hypercellularity, cellular crescents, karyorrhexis/fibrinoid necrosis or subendothelial hyaline deposits. Urine pH was positively correlated with tubular atrophy. There was significantly positive correlation between urine titratable acid and glomerular leukocyte infiltration, whereas urine titratable acid was negatively correlated with CI scores, glomerular sclerosis, tubular atrophy, and interstitial fibrosis. Urine ammonia had significant negative correlations with AI scores, interstitial inflammatory cell infiltration, CI scores, glomerular sclerosis, fibrous crescents, tubular atrophy and interstitial fibrosis.

## Discussion

In this study, a defect of renal tubular function in lupus nephritis was mainly reflected by the impaired urinary acidification. We found that urine ammonia was inversely correlated with SLEDAI-2 K scores, rSLEDAI scores, serum creatinine levels and proteinuria, while it was positively correlated with eGFR. And urine titratable acid was only inversely correlated with rSLEDAI scores and proteinuria. However, the strong association between titratable acid excretion and proteinuria is significant even after adjusting for the chronicity index and eGFR at biopsy. Furthermore, urine ammonia was associated with pathological features of chronicity and tubulointerstitial disease activity. Urine ammonia was a potential reflector of disease activity and tubulointerstitial lesions in lupus nephritis.

Lupus nephritis encompasses diverse patterns of renal disease, including glomerular, tubulointerstitial, and vascular lesions [26]. Growing evidence showed that tubulointerstitial lesions might reflect the severity of lupus nephritis [18, 27, 28]. Y Nozaki et al. found that urinary kidney injury molecule-1 (KIM-1), a specific biomarker for acute tubular damage, was increased in LN and correlated with proteinuria [23]. Several studies have indicated that elevated urinary neutrophil gelatinase-associated lipocalin (NGAL) and monocyte chemoattractant protein-1 (MCP-1) in LN were associated with renal injury indices such as serum creatinine and proteinuria [29–31]. These findings were consistent with our study that urine ammonia was correlated with serum creatinine, eGFR, and proteinuria. This phenomenon can be explained by the assumption that glomerular proteinuria damages tubules, leading to interstitial inflammation and fibrosis [28]. Our results were also consistent with the view that tubular damage was correlated with the abatement of GFR [32]. Moreover, urine ammonia levels were found to be negatively correlated with both SLEDAI-2 K scores and rSLEDAI scores. Similarly, Turnier et al. found that urinary S100A4 levels were elevated in patients with active LN, and levels of urine S100A4 decreased upon disease activity improvement [33]. Another study identified that urinary colony-stimulating factor-1 levels were associated with disease activity, which was a potential biomarker to reflect the onset, recurrence and disease activity of lupus nephritis [27]. These findings supported that urinary biomarkers could serve as sensitive indicators for disease activity in lupus nephritis.

The renal biopsy remains the gold standard for the diagnosis of LN, which has a critical role in guiding therapeutic strategy and predicting prognosis. Y Ding et al. illustrated that urine KIM-1, NGAL, and MCP-1 were sensitive factors for the indication of tubulointerstitial lesions in lupus nephritis, and the combination of NGAL and KIM-1 was identified as a renal prognostic factor [29]. In our study, urine ammonia was negatively correlated with interstitial inflammatory cell infiltration, tubular atrophy, and interstitial fibrosis. And there was a significantly lower trend of urine ammonia in patients with more severe tubulointerstitial lesions. Therefore, our study provided clinical evidence in support of the prior findings that several urinary biomarkers might accurately identity tubulointerstitial damage in LN. Furthermore, active pathological lesions, reflected by cellular crescents and fibrinoid necrosis, foretell adverse disease progression. And chronic lesions also have a high association with disease progression among patients with lupus nephritis [25]. It has been demonstrated that chronic lesions were better than active lesions in reflecting the progressive eGFR decline [3]. In the present study, urine ammonia was negatively correlated with both AI and CI scores, especially several indicators of chronic lesions.

Chronic kidney disease patients with clinically normal acid-base status might have acid-related kidney injury because of enhanced ammonia production by per nephron, which promoting tubulointerstitial fibrosis and further kidney disease progression through intrarenal activation of the alternative pathway of complement [34]. In a cohort study, low urine ammonia excretion was associated with an increased risk of CKD progression [14]. Further studies are needed to explore whether urine ammonia is an influencing factor for lupus nephritis progression.

There are several limitations to the present study. First, this study included a small number of patients. Larger cohorts will be needed to confirm our findings. Second, our study focused on relationships between these urinary markers and lupus nephritis clinical and histopathological features, further studies are required to assess potential effects of urine acidification indicators in recurrence, disease progression and prognosis in lupus nephritis. Third, only some patients in our study underwent the measurements of arterial blood gas tests, renal tubular acidosis in this cohort remained unclear.

## Conclusion

Urine ammonia was associated with clinical and pathological features of chronicity and tubulointerstitial disease activity among patients with lupus nephritis. Furthermore, the strong association between urinary protein and titratable acid excretion at the time of kidney biopsy is significant even after adjusting for the chronicity index and eGFR at biopsy. Future studies can further assess serial measures of urine acidification to determine robustness over time.

## Data Availability

The datasets analyzed during the current study are available from the corresponding author on reasonable request.

## Abbreviations

LN: Lupus nephritis
SLE: Systemic lupus erythematosus
ESRD: End-stage kidney disease
RTA: Renal tubular acidosis
CKD: Chronic kidney disease
GFR: Glomerular filtration rate
BUN: Blood urea nitrogen
C3: Complement 3
ANA: Antinuclear antibodies
anti-dsDNA: Anti-double-stranded DNA
CKD-EPI: Chronic kidney disease epidemiology collaboration
SLEDAI-2 K: SLE disease activity index 2000
rSLEDAI: SLE disease activity index
AI: Activity index
CI: Chronicity index
KIM-1: Kidney injury molecule-1
NGAL: Neutrophil gelatinase-associated lipocalin
MCP-1: Monocyte chemoattractant protein-1
